# Supplementary material for: Histone acetylation orchestrates wound-induced transcriptional activation and cellular reprogramming in Arabidopsis
Source: Commun Biol. 2019 Nov 4;2:404. doi: 10.1038/s42003-019-0646-5 (PMC6828771; doi:10.1038/s42003-019-0646-5)
Supplement: Supplementary file 1 — Supplementary Information [file 42003_2019_646_MOESM1_ESM.pdf]

Supplementary Figures

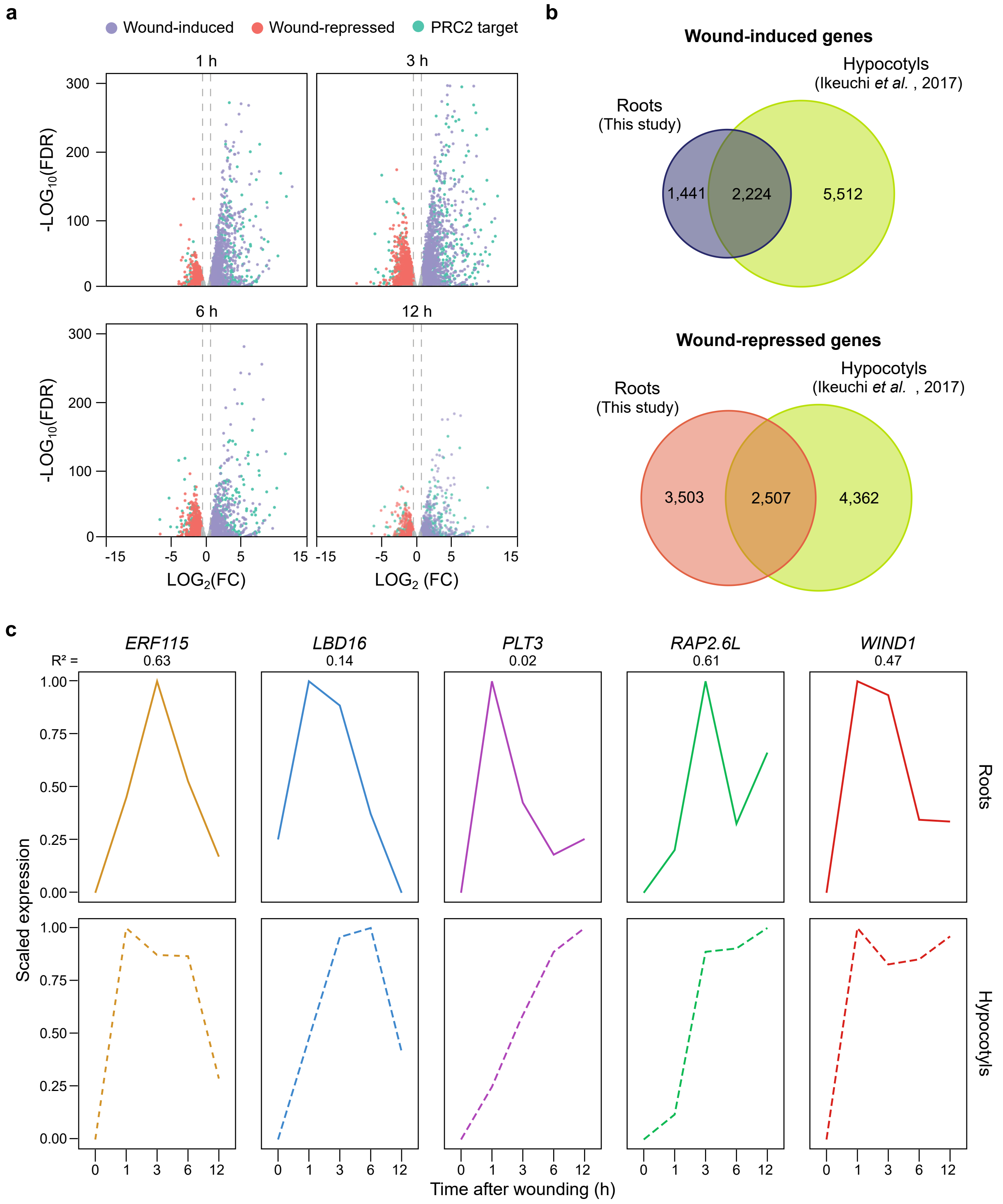

Supplementary Figure 1: Wound-induced transcriptional changes in Arabidopsis roots.

(a) Volcano plots representing the transcriptional changes at 1, 3, 6, and 12 h after wounding. Purple dots mark the genes significantly induced by wounding (FC > 1.5, FDR (edgeR test) < 0.001), and red dots mark the genes significantly repressed by wounding (FC < -1.5, FDR (edgeR test) < 0.001). Turquoise dots represent the genes marked with H3K27me3 before wounding.

(b) Venn-diagram displaying the overlap between wound-induced or wound-repressed genes detected in roots and in hypocotyls[6].

(c) Transcriptional changes of a selection of reprogramming genes in wounded roots and hypocotyls[6]. R^2 values indicate the Pearson correlation coefficient between the expression in roots and hypocotyls.

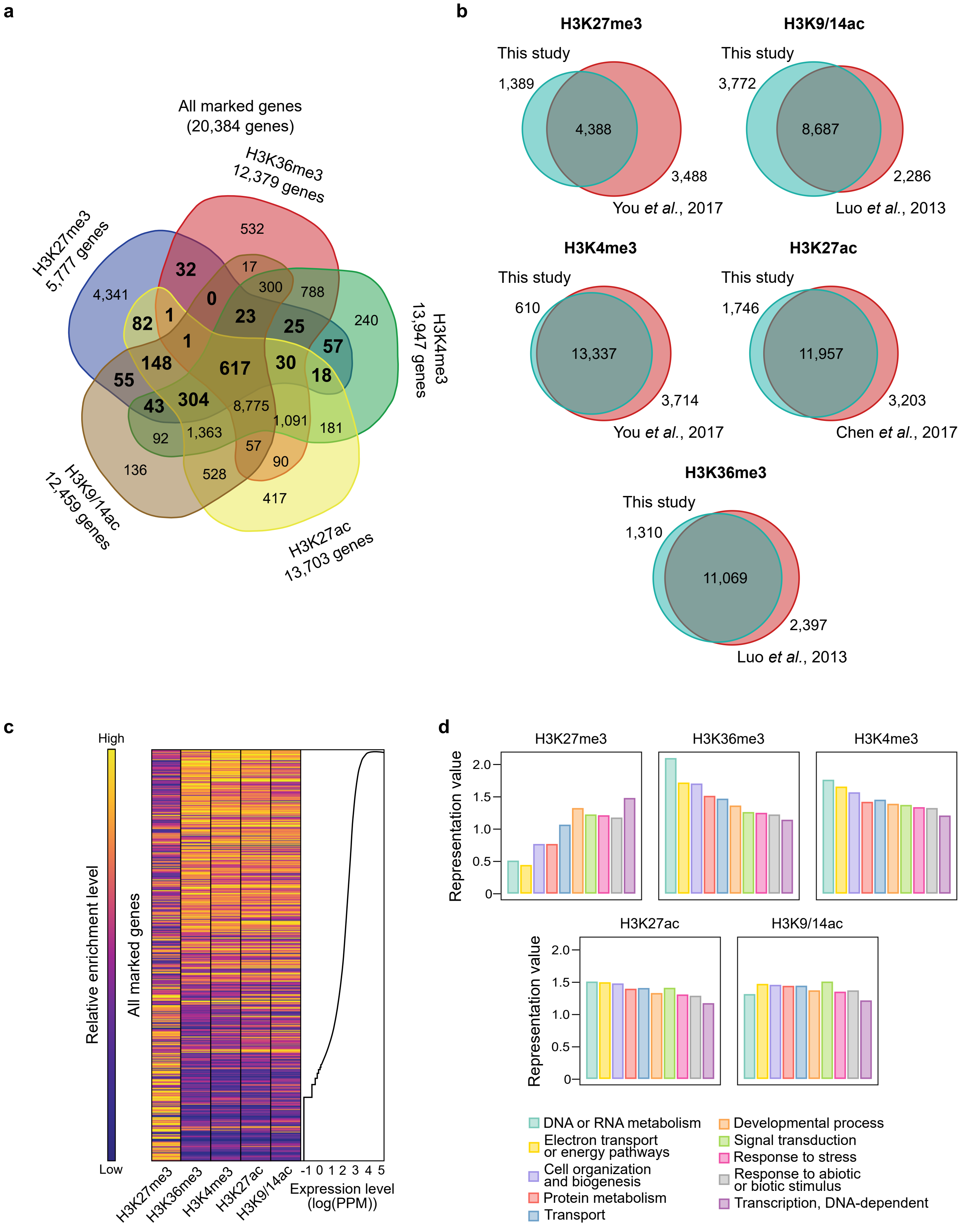

Supplementary Figure 2: Pre-wound deposition of H3K27me3, H3K4me3, H3K9/14ac, H3K27ac and H3K36me3 and gene expression.

(a) Venn diagram representation of the pre-wound states of H3K27me3, H3K36me3, H3K4me3, H3K27ac and H3K9/14 chromatin modifications for the 20,384 genes that possess at least one of these histone marks. The 1,436 genes that possess both repressive H3K27me3 and at least one permissive marks are inscribed in bold.

(b) Venn-diagrams comparing genes detected as marked by H3K27me3, H3K36me3, H3K4me3, H3K27ac or H3K9/14ac in our ChIP-seq dataset with those identified in previous studies[51-53].

(c) Heat map comparing the relative level of pre-wound H3K27me3, H3K36me3, H3K4me3, H3K27ac and H3K9/14ac and gene expression for the 20,384 marked genes. Genes are sorted based on their transcript level before wounding.

(d) Representation of GO categories distribution among genes marked with H3K27me3, H3K36me3, H3K4me3, H3K27ac and H3K9/14ac before wounding.

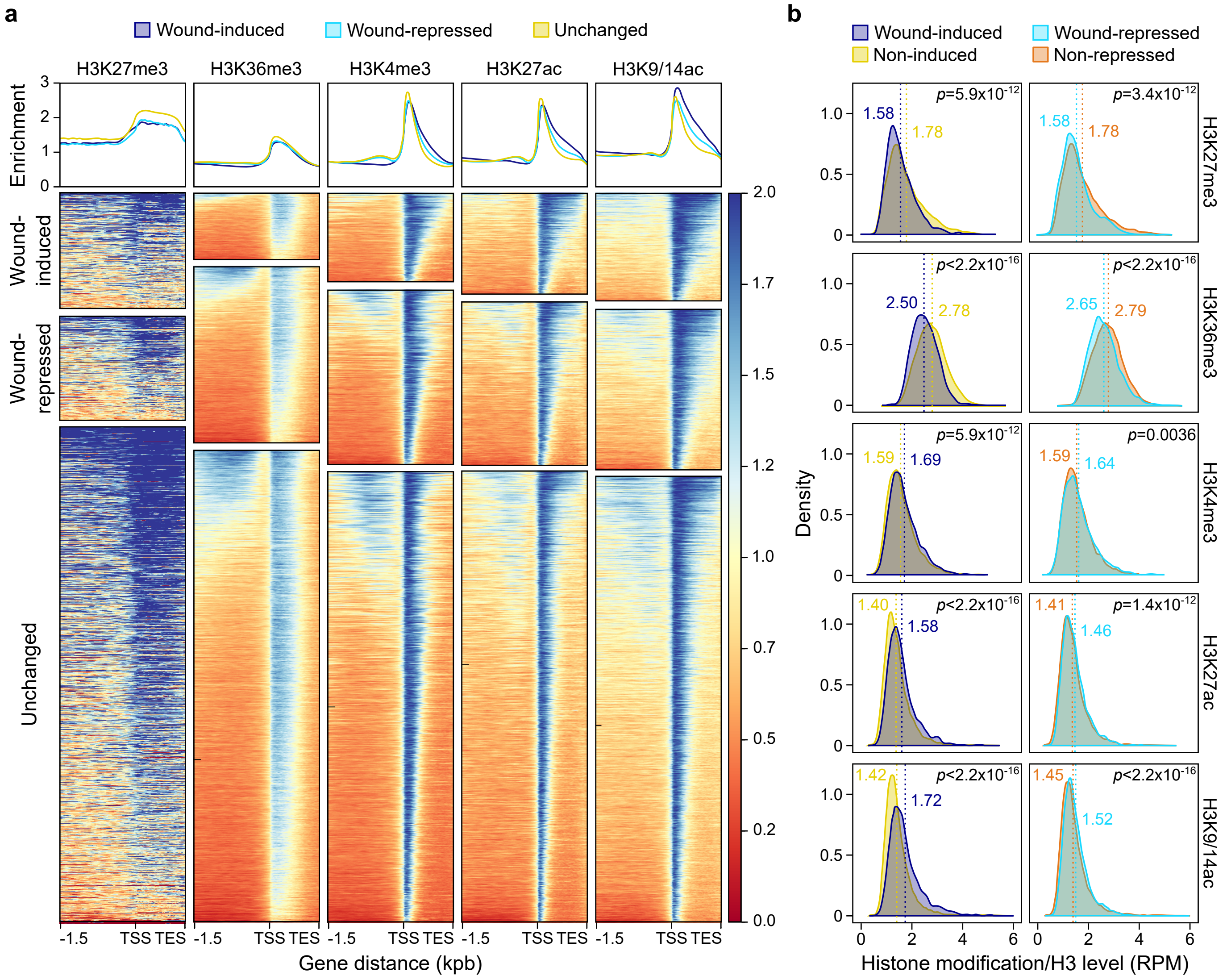

Supplementary Figure 3: Distribution of histone marks for wound-induced, wound-repressed and unchanged genes before wounding.

(a) Heat map showing the ChIP-seq read densities for H3K27me3, H3K36me3, H3K4me3, H3K27ac and H3K9/14ac across the 1.5 kb promoter region and gene body of wound-induced, wound-repressed and unchanged genes. The positions of the transcriptional start site (TSS) and the transcriptional end site (TES) are marked. Line graphs show the average profile of the corresponding histone modification for wound-induced (blue lines), wound-repressed (cyan lines) and non-induced genes (yellow lines).

(b) Density plots comparing the distribution of histone modification levels among (left) wound-induced and non-induced genes and (right) wound-repressed and non-repressed genes. Histone modification levels were calculated across the 1 kb promoter region and gene body of each gene, and corrected for histone H3 abundance. Numbers indicate the mean histone modification levels and the *p* values (Wilcoxon-test).

GO term	Wound-induced Cluster 1	Cluster 2	Cluster 3	Cluster 4	Cluster 5	Cluster 6	Cluster 7	Cluster 8
response to stimulus	1	1	1	1	4	1	NS	>10
response to chemical	2	3	3	3	>10	7	NS	>10
regulation of macromolecule biosynthetic process	3	>10	>10	>10	>10	>10	NS	>10
regulation of nucleobase-containing compound metabolic process	4	>10	>10	>10	>10	>10	NS	>10
regulation of nitrogen compound metabolic process	5	>10	>10	>10	>10	>10	NS	>10
response to organic substance	6	5	6	5	>10	>10	NS	>10
regulation of biosynthetic process	7	>10	>10	>10	>10	>10	NS	>10
regulation of cellular biosynthetic process	8	>10	>10	>10	>10	>10	NS	>10
regulation of cellular metabolic process	9	>10	>10	>10	>10	>10	NS	>10
regulation of primary metabolic process	10	>10	>10	>10	>10	>10	NS	>10
response to chitin	>10	2	4	>10	>10	>10	NS	>10
response to carbohydrate	>10	4	5	>10	>10	>10	NS	>10
response to stress	>10	6	2	2	>10	4	NS	>10
response to wounding	>10	7	>10	>10	>10	>10	NS	>10
response to endogenous stimulus	>10	8	>10	>10	>10	>10	NS	>10
response to water deprivation	>10	9	>10	>10	>10	>10	NS	>10
response to water	>10	10	>10	>10	>10	>10	NS	>10
defense response	>10	>10	7	4	>10	9	NS	>10
response to biotic stimulus	>10	>10	8	>10	>10	5	NS	>10
cellular nitrogen compound metabolic process	>10	>10	9	6	>10	>10	NS	>10
response to other organism	>10	>10	10	>10	>10	2	NS	>10
protein phosphorylation	>10	>10	>10	9	>10	>10	NS	>10
multi-organism process	>10	>10	>10	7	>10	3	NS	>10
immune system process	>10	>10	>10	8	>10	>10	NS	>10
immune response	>10	>10	>10	10	>10	>10	NS	>10
secondary metabolic process	>10	>10	>10	>10	>10	8	NS	1
response to drug	>10	>10	>10	>10	1	>10	NS	>10
drug transmembrane transport	>10	>10	>10	>10	2	>10	NS	>10
drug transport	>10	>10	>10	>10	3	>10	NS	>10
glucosinolate metabolic process	>10	>10	>10	>10	5	>10	NS	>10
glycosinolate metabolic process	>10	>10	>10	>10	6	>10	NS	>10
glycoside metabolic process	>10	>10	>10	>10	7	>10	NS	7
S-glycoside metabolic process	>10	>10	>10	>10	8	>10	NS	>10
cellular carbohydrate metabolic process	>10	>10	>10	>10	9	>10	NS	>10
sulfur compound metabolic process	>10	>10	>10	>10	10	>10	NS	>10
response to bacterium	>10	>10	>10	>10	>10	6	NS	>10
defense response to bacterium	>10	>10	>10	>10	>10	10	NS	>10
glucosinolate biosynthetic process	>10	>10	>10	>10	>10	>10	NS	2
S-glycoside biosynthetic process	>10	>10	>10	>10	>10	>10	NS	3
glycosinolate biosynthetic process	>10	>10	>10	>10	>10	>10	NS	4
glycoside biosynthetic process	>10	>10	>10	>10	>10	>10	NS	5
cell redix homeostasis	>10	>10	>10	>10	>10	>10	NS	6
cellular aromatic compound metabolic process	>10	>10	>10	>10	>10	>10	NS	8
response to UV	>10	>10	>10	>10	>10	>10	NS	9
response to light stimulus	>10	>10	>10	>10	>10	>10	NS	10

Supplementary Figure 4: GO analysis on wound-induced genes.
List of the top 10 GO terms enriched among genes constituting the induced clusters 1 to 8, as depicted in Figure 2a. The color gradient represents the rank of significance for the enrichment of each GO term.

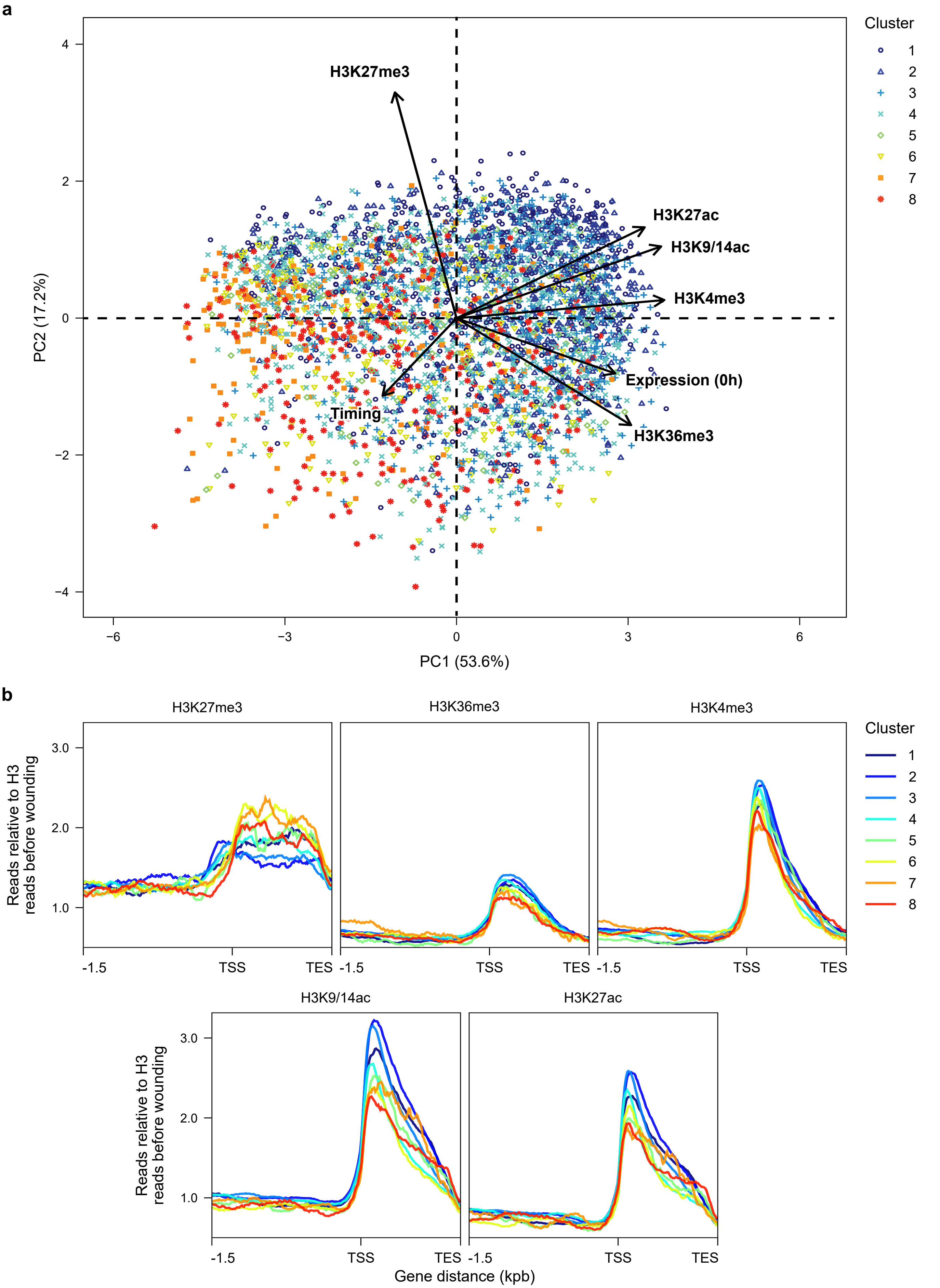

Supplementary Figure 5: Correlation between pre-wound histone modification and timing of gene expression. **(a)** Principal Component Analysis (PCA) plot showing the multivariate variation among wound-induced genes in terms of presence in clusters 1 to 8 (timing), levels of expression before wounding (expression (0h)) and the levels of pre-wound H3K27me3, H3K36me3, H3K4me3, H3K27ac and H3K9/14ac. Arrows indicate the direction and strength of each variable to the overall distribution. Colored symbols correspond to the K-means clusters defined in Figure 2a. **(b)** Metaplots showing the average ChIP-seq read density distribution for H3K27me3, H3K36me3, H3K4me3, H3K27ac

and H3K9/14ac across the 1.5 kb promoter region and gene body for genes in clusters 1 to 8. The positions of the transcriptional start site (TSS) and the transcriptional end site (TES) are marked.

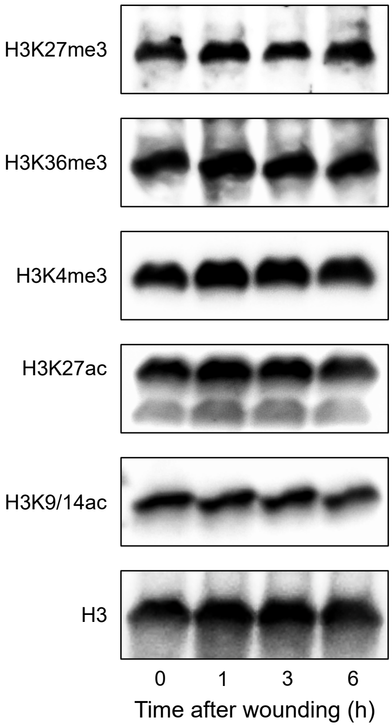

Supplementary Figure 6: Western blot analysis of H3K27me3, H3K36me3, H3K4me3, H3K27ac and H3K9/14ac levels at 0, 1, 3, 6 h after wounding.

The enrichment of histone marks is shown relative to histone H3 which is used as a loading control.

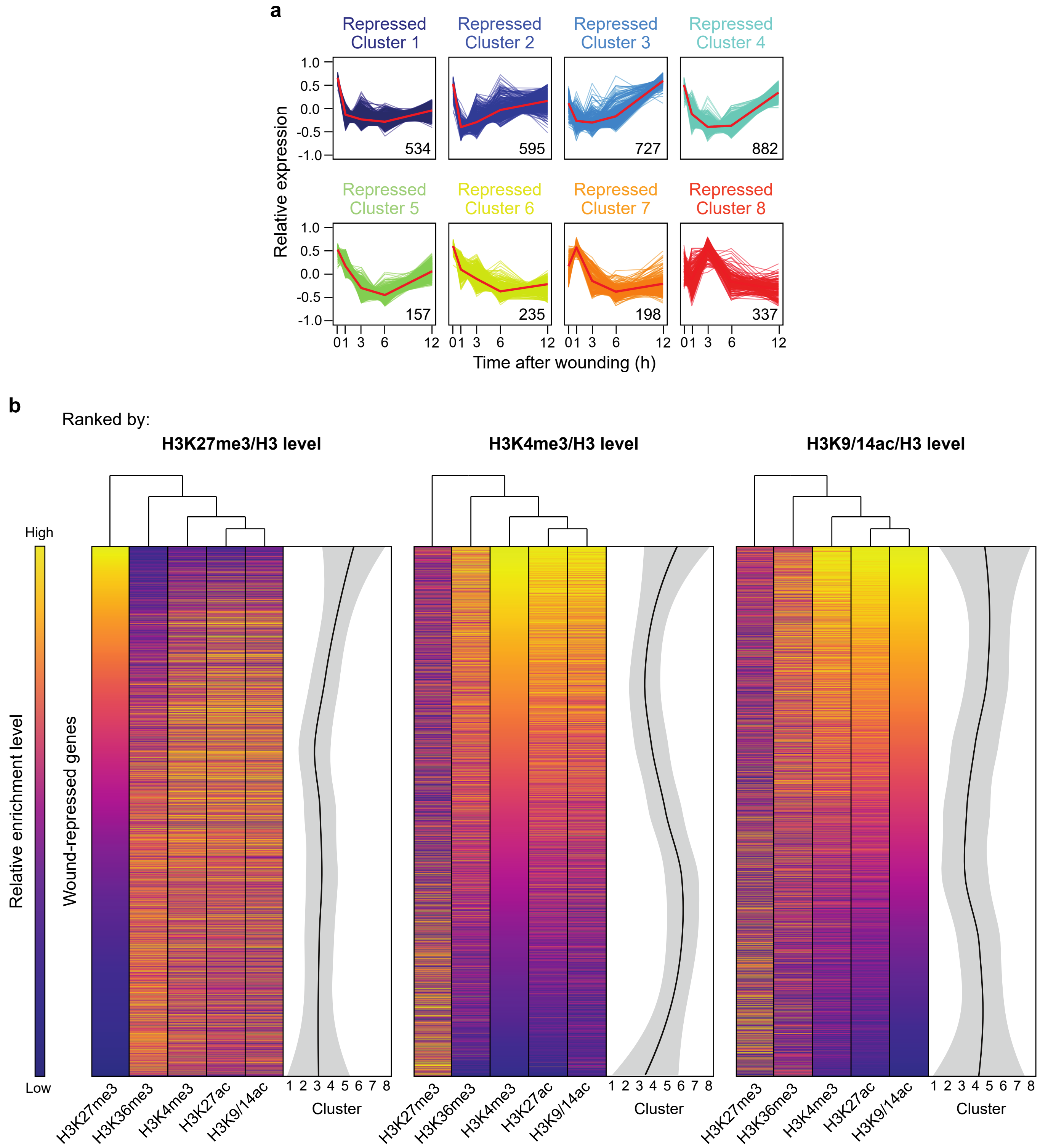

Supplementary Figure 7: Pre-wound histone marking levels associated with timing of transcriptional repression.

(a) K-means clustering of genes significantly repressed by wounding (FC < -1.5, FDR (edgeR test) < 0.001, n = 6,010 genes). Clusters are ordered from most rapidly (repressed cluster 1) to most slowly (repressed cluster 8) repressed genes. The number of genes included in each cluster is indicated and red lines show the average level of their expression.

(b) Heat map-based comparison of the relative enrichment levels of pre-wound H3K27me3, H3K36me3, H3K4me3, H3K27ac and H3K9/14ac and the timing of transcriptional repression after wounding, using a LOESS regression. Genes are sorted based on their relative enrichment levels of pre-wound H3K27me3, H3K4me3 or H3K9/14ac.

GO term	Wound-repressed Cluster 1	Cluster 2	Cluster 3	Cluster 4	Cluster 5	Cluster 6	Cluster 7	Cluster 8
cellular carbohydrate metabolic process	1	>10	>10	>10	>10	>10	>10	>10
alcohol metabolic process	2	>10	>10	>10	>10	>10	>10	>10
carbohydrate metabolic process	3	>10	>10	>10	>10	>10	>10	>10
alcohol biosynthetic process	4	>10	>10	>10	>10	>10	>10	>10
dolichol metabolic process	5	>10	>10	>10	>10	>10	>10	>10
dolichol biosynthetic process	6	>10	>10	>10	>10	>10	>10	>10
developmental growth involved in morphogenesis	7	>10	>10	>10	>10	>10	>10	>10
unidimensional cell growth	8	>10	>10	>10	>10	>10	>10	>10
hexose metabolic process	9	>10	>10	>10	>10	>10	>10	>10
prenol biosynthetic process	10	>10	>10	>10	>10	>10	>10	>10
plant-type cell wall organization	>10	1	>10	>10	>10	>10	>10	>10
peptide transport	>10	2	>10	>10	>10	>10	>10	>10
oligopeptide transport	>10	3	>10	>10	>10	>10	>10	>10
secondary metabolic process	>10	>10	1	>10	>10	>10	>10	>10
cellular aromatic compound metabolic process	>10	>10	2	>10	>10	>10	>10	>10
phenylpropanoid metabolic process	>10	>10	3	>10	>10	>10	>10	>10
flavonoid biosynthetic process	>10	>10	4	>10	>10	>10	>10	>10
response to light stimulus	>10	>10	5	>10	>10	>10	>10	>10
response to radiation	>10	>10	6	>10	>10	>10	>10	>10
flavonoid metabolic process	>10	>10	7	>10	>10	>10	>10	>10
phenylpropanoid biosynthetic process	>10	>10	8	>10	>10	>10	>10	>10
pigment biosynthetic process	>10	>10	9	>10	>10	>10	>10	>10
glucosinolate biosynthetic process	>10	>10	10	>10	>10	>10	>10	>10
microtubule-based process	>10	>10	>10	1	7	>10	>10	>10
cell cycle	>10	>10	>10	2	4	>10	>10	>10
microtubule-based movement	>10	>10	>10	3	>10	>10	>10	>10
post-embryonic development	>10	>10	>10	4	8	>10	>10	>10
cell cycle process	>10	>10	>10	5	>10	>10	>10	>10
cell cycle phase	>10	>10	>10	6	>10	>10	>10	>10
regulation of organelle organization	>10	>10	>10	7	>10	>10	>10	>10
cellular nitrogen compound metabolic process	>10	>10	>10	8	>10	>10	>10	>10
cytoskeleton organization	>10	>10	>10	9	>10	>10	>10	>10
organelle organization	>10	>10	>10	10	>10	>10	>10	>10
DNA metabolic process	>10	>10	>10	>10	1	>10	>10	>10
DNA replication	>10	>10	>10	>10	2	>10	>10	>10
DNA-dependent DNA replication	>10	>10	>10	>10	3	>10	>10	>10
cellular component biogenesis	>10	>10	>10	>10	5	>10	>10	>10
DNA unwinding involved in DNA replication	>10	>10	>10	>10	6	>10	>10	>10
regulation of cell cycle	>10	>10	>10	>10	9	>10	>10	>10
DNA duplex unwinding	>10	>10	>10	>10	10	>10	>10	>10
translation	>10	>10	>10	>10	>10	1	>10	>10
cellular protein metabolic process	>10	>10	>10	>10	>10	2	>10	>10
macromolecule biosynthetic process	>10	>10	>10	>10	>10	3	>10	>10
cellular macromolecule biosynthetic process	>10	>10	>10	>10	>10	4	>10	>10
biosynthetic process	>10	>10	>10	>10	>10	5	>10	>10
cellular biosynthetic process	>10	>10	>10	>10	>10	6	>10	>10
protein metabolic process	>10	>10	>10	>10	>10	7	>10	>10
ribosome biogenesis	>10	>10	>10	>10	>10	8	>10	>10
ribonucleoprotein complex biogenesis	>10	>10	>10	>10	>10	9	>10	>10
gene expression	>10	>10	>10	>10	>10	10	>10	>10
nucleosome organization	>10	>10	>10	>10	>10	>10	1	>10
chromatin assembly	>10	>10	>10	>10	>10	>10	2	>10
nucleosome assembly	>10	>10	>10	>10	>10	>10	3	>10
protein-DNA complex assembly	>10	>10	>10	>10	>10	>10	4	>10
DNA packaging	>10	>10	>10	>10	>10	>10	5	>10
chromatin assembly or disassembly	>10	>10	>10	>10	>10	>10	6	>10
response to chemical	>10	>10	>10	>10	>10	>10	7	1
chromatin organization	>10	>10	>10	>10	>10	>10	8	>10
response to organic substance	>10	>10	>10	>10	>10	>10	9	2
regulation of transcription, DNA-templated	>10	>10	>10	>10	>10	>10	10	>10
response to hormone	>10	>10	>10	>10	>10	>10	>10	3
response to auxin	>10	>10	>10	>10	>10	>10	>10	4
response to endogenous stimulus	>10	>10	>10	>10	>10	>10	>10	5
response to stimulus	>10	>10	>10	>10	>10	>10	>10	6
response to abiotic stimulus	>10	>10	>10	>10	>10	>10	>10	7
response to oxidative stress	>10	>10	>10	>10	>10	>10	>10	8
response to temperature stimulus	>10	>10	>10	>10	>10	>10	>10	9

Supplementary Figure 8: GO analysis on wound-repressed genes.
List of the top 10 GO terms enriched among genes constituting the repressed clusters 1 to 8, as depicted in Figure S7a. The color gradient represents the rank of significance for the enrichment of each GO term.

pre-wound H3K9/14ac	-	-	+	+	-	+	-	+
post-wound gain in H3K9/14ac	-	+	-	+	-	-	+	+
post-wound gain in H3K4me3	-	-	-	-	+	+	+	+

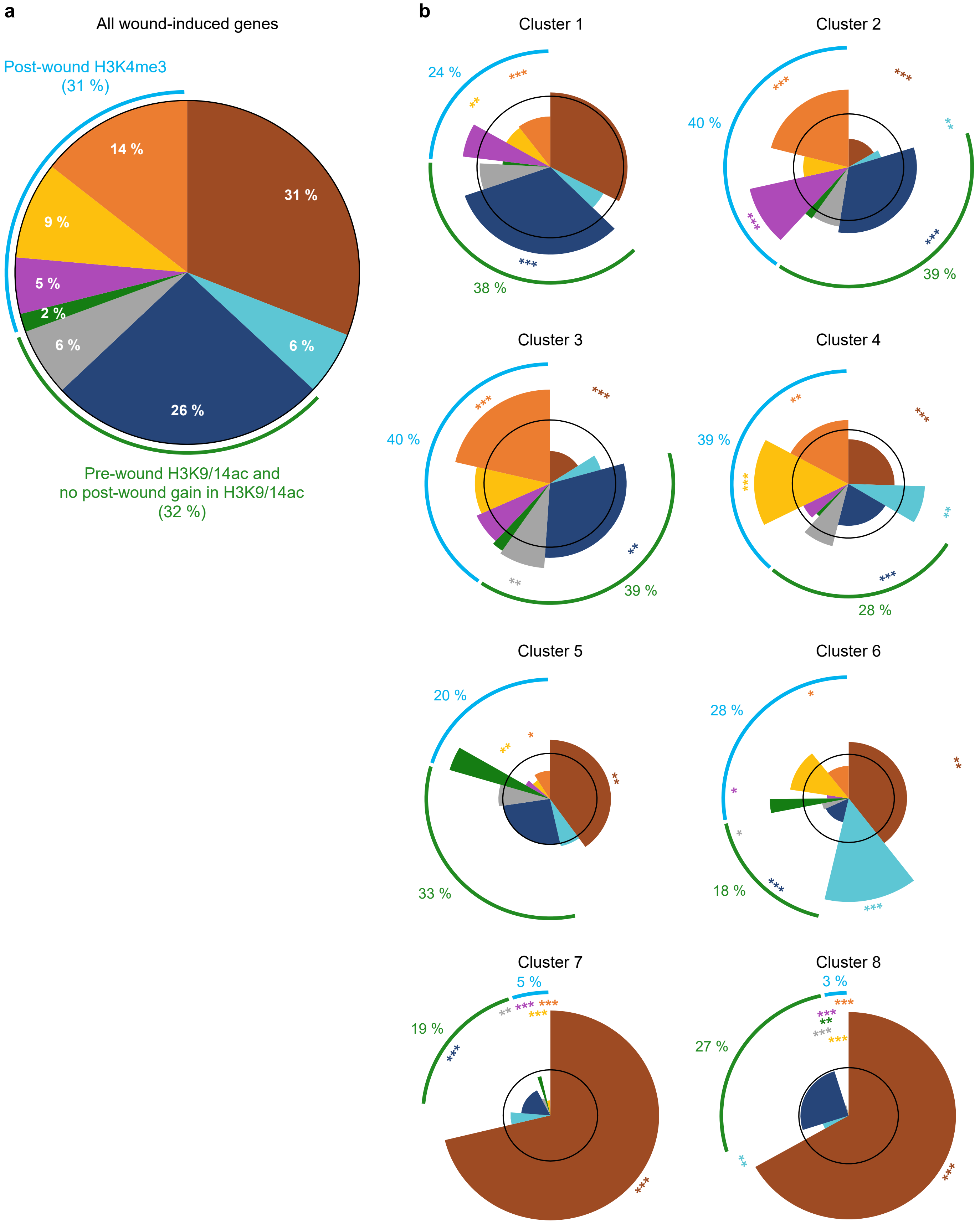

Supplementary Figure 9: Wound-induced accumulation of H3K4me3 is associated with slower transcriptional activation.

(a) Pie chart representing the percentages of genes associated with pre-wound H3K9/K14ac, post-wound H3K9/14ac and/or H3K4me3 among all 3,665 wound-induced genes. Genes are grouped based on their association with post-wound H3K4me3

(b) Spie chart representing the percentage of genes associated with pre-wound H3K9/K14ac, post-wound H3K9/14ac

and/or post-wound H3K4me3 among genes within clusters 1 to 8. Genes are grouped based on their association with post-wound H3K4me3. The radius of the wedges corresponds to the representation factor (hypergeometric test) of the epigenetic category in the cluster compared to its representation among all wound-induced genes. The black circle corresponds to a representation factor of 1 so that wedges inside the circle describe an underrepresented category and wedges that extend beyond the circle describe an overrepresented category. $^{*}p < 0.05$, $^{**}p < 0.01$, $^{***}p < 0.001$ (hypergeometric test).

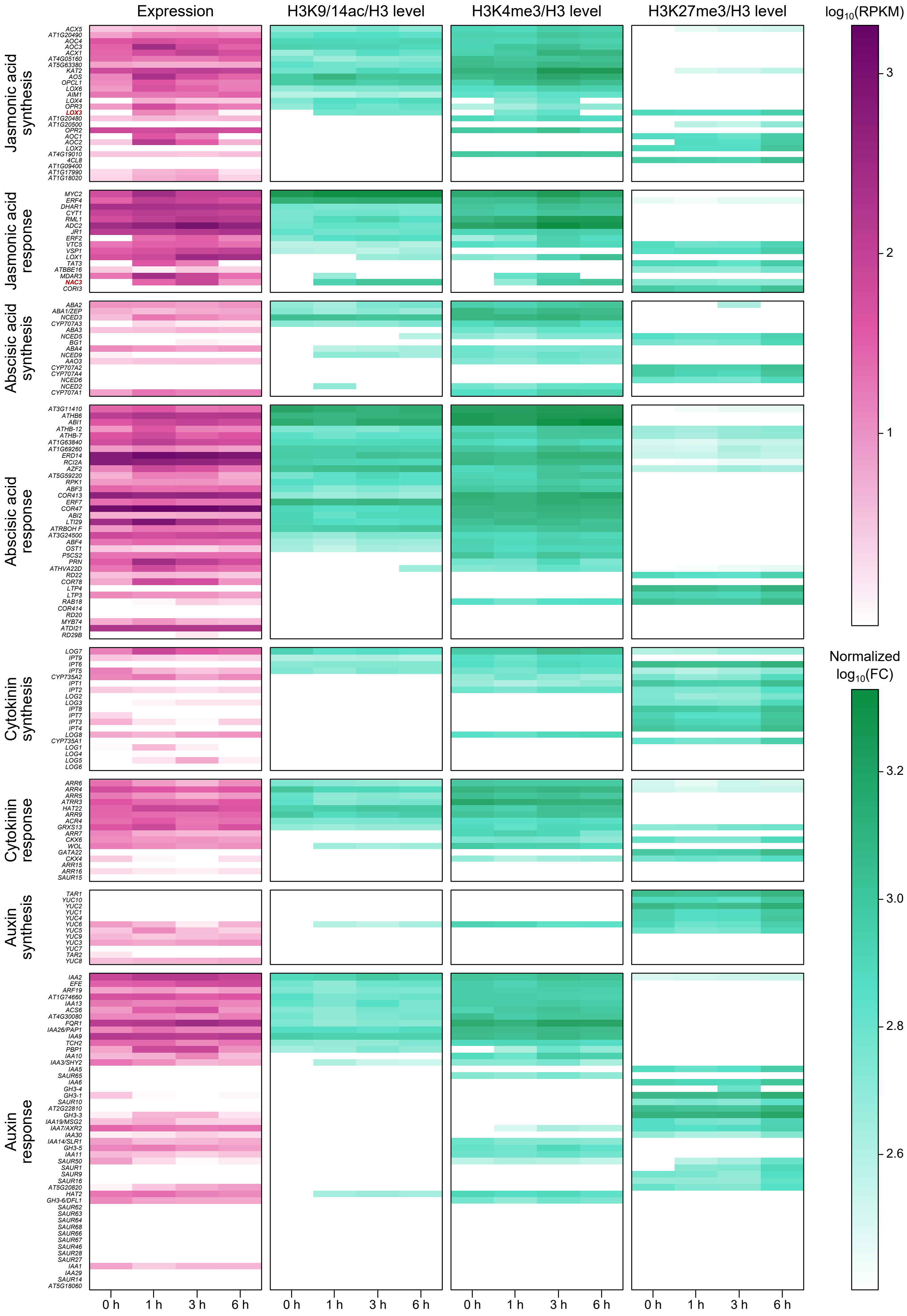

Supplementary Figure 10: Wound-induced changes of gene expression and histone modification for hormone biosynthesis and signalling genes.

Genes involved in synthesis and signaling of jasmonic acid, abscisic acid, cytokinin and auxin were selected following Ikeuchi et al (2017). For histone marks, the $\log_{10}$(FC) values were normalized using the MAnorm method[78].

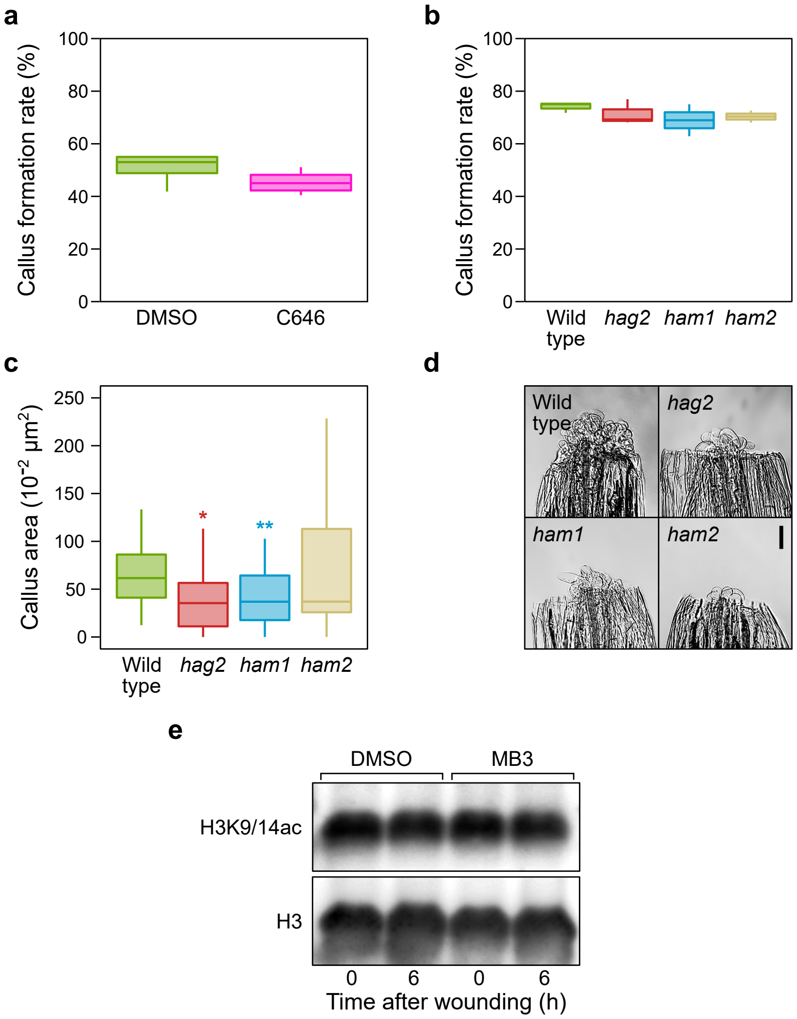

Supplementary Figure 11: Inhibition of HAG2, HAM1, HAM2 or CBP-mediated histone acetylation does not block wound-induced callus formation.

(a) Percentage of Arabidopsis hypocotyls that form callus at wound sites. Plants were treated with DMSO or 100 µM C646 from 24 h before wounding. More than 40 plants were evaluated per replicate and error bars show the standard deviation (n = 4 independent experiments). Significant differences were tested by a Student's t-test.

(b) Percentage of hypocotyls that form callus at wound sites in Col-0, *hag2*, *ham1* and *ham2*. More than 40 plants were evaluated per experiment and error bars show the standard deviation (n = 4 independent experiments). Significant differences were tested by a Student's t-test.

(c) Quantitative analysis of callus area in Col-0, *hag2*, *ham1* and *ham2* hypocotyls. Error bars show the standard deviation (n > 100 biologically independent samples). $*p < 0.05$, $**p < 0.01$, (Student's t-test).

(d) Wound-induced callus in Col-0, *hag2*, *ham1* and *ham2* hypocotyls. Scale bar = 100 µm.

(e) Western blot analysis of H3K9/14ac levels in plants treated with DMSO or 100 µM MB3. The enrichment of H3K9/14ac is shown relative to histone H3 which is used as a loading control.

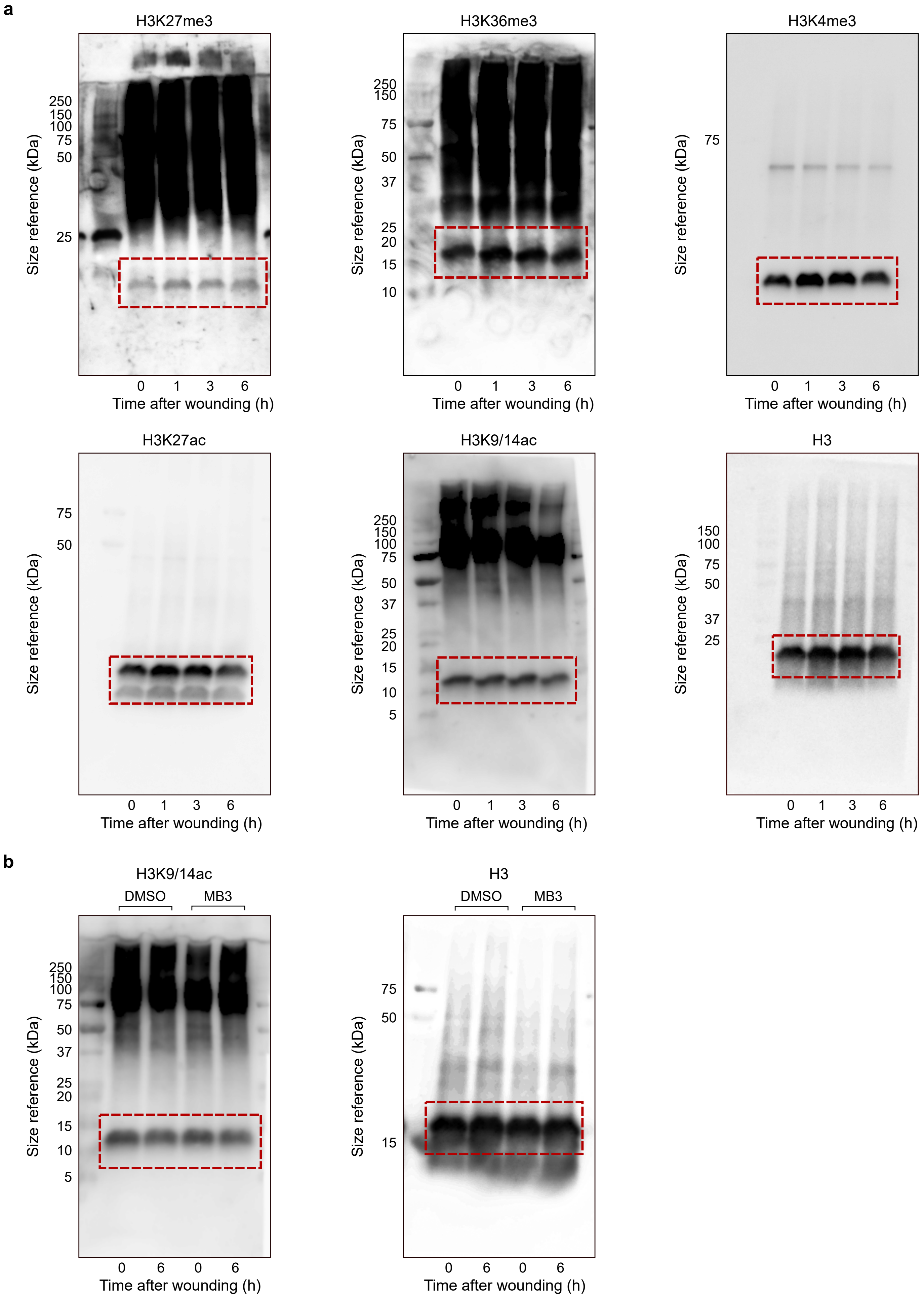

Supplementary Figure 12: Full images of western blot analysis for histone modification

(a) Western blot analysis of H3K27me3, H3K36me3, H3K4me3, H3K27ac and H3K9/14ac levels at 0, 1, 3, 6 h after wounding.

(b) Western blot analysis of H3K9/14ac levels in plants treated with DMSO or 100 μM MB3.
