# Supplementary material for: Histone acetylation orchestrates wound-induced transcriptional activation and cellular reprogramming in Arabidopsis
Source: Commun Biol. 2019 Nov 4;2:404. doi: 10.1038/s42003-019-0646-5 (PMC6828771; doi:10.1038/s42003-019-0646-5)
Supplement: Supplementary file 2 — Description of additional supplementary items [file 42003_2019_646_MOESM2_ESM.docx]

**Description of additional supplementary items – Rymen et al (2019)**

**Supplementary Data**

**Supplementary Data 1: List of genes induced (S1a) or repressed (S1b) by wounding in Arabidopsis roots.**

**Supplementary Data 2: List of genes associated with H3K27me3, H3K9/14ac, H3K27ac, H3K4me3, and H3K36me3 prior to wounding.**

**Supplementary Data 3: List of genes with modified H3K27me3, H3K9/14ac, H3K27ac, H3K4me3, and/or H3K36me3 levels after wounding.**

**Supplementary Data 4: List of wound-induced genes that lose H3K27me3 or gain H3K9/14ac, H3K27ac, H3K4me3, and H3K36me3 after wounding.**

**Supplementary Data 5: List of wound-repressed genes that gain H3K27me3 or lose H3K9/14ac, H3K27ac, H3K4me3, and H3K36me3 after wounding.**

**Supplementary Data 6: List of genes with modified levels of H3K9/14ac or H3K4me3 after exposure to 100 µM MB3 at least at one time point between 0 h and 6 h after wounding.**

**Supplementary Data 7: List of genes down-regulated (FC < -1.5) by exposure to 100 µM MB3 at least at one time point between 0 h and 6 h after wounding.**

**Supplementary Data 8: Source Data used for graphs and charts in main Figures**
