# Supplementary material for: Histone acetylation orchestrates wound-induced transcriptional activation and cellular reprogramming in Arabidopsis
Source: Commun Biol. 2019 Nov 4;2:404. doi: 10.1038/s42003-019-0646-5 (PMC6828771; doi:10.1038/s42003-019-0646-5)
Supplement: Supplementary file 11 — Reporting Summary [file 42003_2019_646_MOESM11_ESM.pdf]

# nature research

Corresponding author(s): Bart Rymen, Keiko Sugimoto

Last updated by author(s): Sep 26, 2019

# Reporting Summary

Nature Research wishes to improve the reproducibility of the work that we publish. This form provides structure for consistency and transparency in reporting. For further information on Nature Research policies, see Authors & Referees and the Editorial Policy Checklist.

## Statistics

For all statistical analyses, confirm that the following items are present in the figure legend, table legend, main text, or Methods section.

| n/a | Confirmed | |
|---|---|---|
| ☐ | ☒ | The exact sample size ($n$) for each experimental group/condition, given as a discrete number and unit of measurement |
| ☐ | ☒ | A statement on whether measurements were taken from distinct samples or whether the same sample was measured repeatedly |
| ☐ | ☒ | The statistical test(s) used AND whether they are one- or two-sided<br>*Only common tests should be described solely by name; describe more complex techniques in the Methods section.* |
| ☐ | ☒ | A description of all covariates tested |
| ☐ | ☒ | A description of any assumptions or corrections, such as tests of normality and adjustment for multiple comparisons |
| ☐ | ☒ | A full description of the statistical parameters including central tendency (e.g. means) or other basic estimates (e.g. regression coefficient) AND variation (e.g. standard deviation) or associated estimates of uncertainty (e.g. confidence intervals) |
| ☐ | ☒ | For null hypothesis testing, the test statistic (e.g. $F$, $t$, $r$) with confidence intervals, effect sizes, degrees of freedom and $P$ value noted<br>*Give P values as exact values whenever suitable.* |
| ☒ | ☐ | For Bayesian analysis, information on the choice of priors and Markov chain Monte Carlo settings |
| ☒ | ☐ | For hierarchical and complex designs, identification of the appropriate level for tests and full reporting of outcomes |
| ☒ | ☐ | Estimates of effect sizes (e.g. Cohen's $d$, Pearson's $r$), indicating how they were calculated |

*Our web collection on statistics for biologists contains articles on many of the points above.*

## Software and code

Policy information about availability of computer code

| | |
|---|---|
| Data collection | Mapping was done by Bowtie<br>Peak calling by MACS2<br>Further processing was done using BEDTools suite (BamtoBed, coverage, closest) |
| Data analysis | Data was analyzed using R-Studio Version 1.1.456, using the following packages: factoMiner, faxctoextra, ggsignif, ggplot2, superheat, and ggpubr and deeptools 3.1.2 on Python 2.7.15 |

For manuscripts utilizing custom algorithms or software that are central to the research but not yet described in published literature, software must be made available to editors/reviewers. We strongly encourage code deposition in a community repository (e.g. GitHub). See the Nature Research guidelines for submitting code & software for further information.

## Data

Policy information about availability of data

All manuscripts must include a data availability statement. This statement should provide the following information, where applicable:

- Accession codes, unique identifiers, or web links for publicly available datasets
- A list of figures that have associated raw data
- A description of any restrictions on data availability

All raw data is either provided in the submission and for RNA-seq and ChiP-seq raw unprocessed data was deposited to ArrayExpress.

E-MTAB-7612; E-MTAB-7611; E-MTAB-7609; E-MTAB-8265

# Field-specific reporting

Please select the one below that is the best fit for your research. If you are not sure, read the appropriate sections before making your selection.

☒ Life sciences ☐ Behavioural & social sciences ☐ Ecological, evolutionary & environmental sciences

For a reference copy of the document with all sections, see nature.com/documents/nr-reporting-summary-flat.pdf

# Life sciences study design

All studies must disclose on these points even when the disclosure is negative.

| | |
|---|---|
| Sample size | Standard sample size for RNA-seq was used (triplicate of pooled plants (over 300 individuals per rep), for the main ChIP-seq the reproducibility was high enough to make relevant conclusions based on duplicate experiments, the ChIP-Seq to confirm MB3 is a Histone acetyltransferase inhibitor, we performed a single rep at 3 time points after application. For phenotypic analysis, over 40 individual plants per treatment were evaluated, standardized in previous published wound- regeneration assays. Western blots were performed at least in duplicate on pooled samples of over 300 individual plants. |
| Data exclusions | No data was excluded from the analysis. |
| Replication | All data shown is based on multiple biologically independent replicates. For the phenotopic evaluation analysis, we performed all experiments at least 3 times. For RNA seq (three replicates) and main ChIP-seq analysis (duplicates), ChIP-Seq to confirm MB3 is a Histone acetyltransferase inhibitor, we performed a single rep at 3 time points after application. For all experiments plants were grown independently and samples were from pooled over 300 individuals. |
| Randomization | Plants were always grown in vitro on multiple petridishes per treatment to avoid plate effects. In addition were plates placed randomized across treatments in the growth chambers. |
| Blinding | Investigators were not blinded during sample preparations. This is impractical, since the samples had to be sampled very fast after treatment and were further processed by the same researcher. During sample collection, however, always two researchers were present. Sequence services were not aware of type of samples or treatment prior to sample processing. For phenotypic evaluation, tests were repeated by at least two different researchers. |

# Reporting for specific materials, systems and methods

We require information from authors about some types of materials, experimental systems and methods used in many studies. Here, indicate whether each material, system or method listed is relevant to your study. If you are not sure if a list item applies to your research, read the appropriate section before selecting a response.

## Materials & experimental systems

| n/a | Involved in the study | |
|---|---|---|
| ☐ | ☒ | Antibodies |
| ☒ | ☐ | Eukaryotic cell lines |
| ☒ | ☐ | Palaeontology |
| ☒ | ☐ | Animals and other organisms |
| ☒ | ☐ | Human research participants |
| ☒ | ☐ | Clinical data |

## Methods

| n/a | Involved in the study | |
|---|---|---|
| ☐ | ☒ | ChIP-seq |
| ☒ | ☐ | Flow cytometry |
| ☒ | ☐ | MRI-based neuroimaging |

## Antibodies

| | |
|---|---|
| Antibodies used | H3 (ab1791; Abcam), H3K27me3 (07-449; Millipore), H3K4me3 (ab8580; Abcam), H3K36me3 (ab9050; Abcam), H3K9/14ac (06-599, Millipore) and H3K27ac (ab4729, Abcam). |
| Validation | All antibodies used were previously used in published data collection for Arabidopsis thaliana. In addition we performed western blots to evaluate if we detected the expected size of protein. |

## ChIP-seq

### Data deposition

☒ Confirm that both raw and final processed data have been deposited in a public database such as GEO.

☒ Confirm that you have deposited or provided access to graph files (e.g. BED files) for the called peaks.

Data access links
*May remain private before publication.*

The main ChIP seq experiment, evaluating chromatin dynamics after wounding is submitted under E-MTAB-7611

The second ChIP-SEQ dataset evaluating MB3 as a histone acetyltransferase as an inhibitor was submitted under E-MTAB-8265

Files in database submission

For the wounding data set (E-MTAB-7611) the following files were uploaded:

H3_0h.bam
H3_0h_rep1_S1_L002_R1_001.fastq.gz
H3_0h_rep1_S1_L003_R1_001.fastq.gz
H3_0h_rep1_S1_L004_R1_001.fastq.gz
H3_0h_Rep2.bam
H3_0h_rep2_S1_L001_R1_001.fastq.gz
H3_0h_rep2_S1_L002_R1_001.fastq.gz
H3_0h_rep2_S1_L003_R1_001.fastq.gz
H3_0h_rep2_S1_L004_R1_001.fastq.gz
H3_1h.bam
H3_1h_rep1_S16_L001_R1_001.fastq.gz
H3_1h_rep1_S16_L002_R1_001.fastq.gz
H3_1h_rep1_S16_L003_R1_001.fastq.gz
H3_1h_rep1_S16_L004_R1_001.fastq.gz
H3_1h_Rep2.bam
H3_1h_rep2_S3_L001_R1_001.fastq.gz
H3_1h_rep2_S3_L002_R1_001.fastq.gz
H3_1h_rep2_S3_L003_R1_001.fastq.gz
H3_1h_rep2_S3_L004_R1_001.fastq.gz
H3_3h.bam
H3_3h_rep1_S19_L001_R1_001.fastq.gz
H3_3h_rep1_S19_L002_R1_001.fastq.gz
H3_3h_rep1_S19_L003_R1_001.fastq.gz
H3_3h_rep1_S19_L004_R1_001.fastq.gz
H3_3h_Rep2.bam
H3_3h_rep2_S2_L001_R1_001.fastq.gz
H3_3h_rep2_S2_L002_R1_001.fastq.gz
H3_3h_rep2_S2_L003_R1_001.fastq.gz
H3_3h_rep2_S2_L004_R1_001.fastq.gz
H3_6h.bam
H3_6h_rep1_S22_L001_R1_001.fastq.gz
H3_6h_rep1_S22_L002_R1_001.fastq.gz
H3_6h_rep1_S22_L003_R1_001.fastq.gz
H3_6h_rep1_S22_L004_R1_001.fastq.gz
H3_6h_Rep2.bam
H3_6h_rep2_S4_L001_R1_001.fastq.gz
H3_6h_rep2_S4_L002_R1_001.fastq.gz
H3_6h_rep2_S4_L003_R1_001.fastq.gz
H3_6h_rep2_S4_L004_R1_001.fastq.gz
H3K14ac_0h.bam
H3K14ac_0h_Peaks.bed
H3K14ac_0h_rep1.bw
H3K14ac_0h_rep1_S10_L001_R1_001.fastq.gz
H3K14ac_0h_rep1_S10_L002_R1_001.fastq.gz
H3K14ac_0h_rep1_S10_L003_R1_001.fastq.gz
H3K14ac_0h_rep1_S10_L004_R1_001.fastq.gz
H3K14ac_0h_Rep2.bam
H3K14ac_0h_rep2.bw
H3K14ac_0h_rep2_S35_L001_R1_001.fastq.gz
H3K14ac_0h_rep2_S35_L002_R1_001.fastq.gz
H3K14ac_0h_rep2_S35_L003_R1_001.fastq.gz
H3K14ac_0h_rep2_S35_L004_R1_001.fastq.gz
H3K14ac_1h.bam
H3K14ac_1h_peaks.bed
H3K14ac_1h_rep1.bw
H3K14ac_1h_rep1_S11_L001_R1_001.fastq.gz
H3K14ac_1h_rep1_S11_L002_R1_001.fastq.gz
H3K14ac_1h_rep1_S11_L003_R1_001.fastq.gz
H3K14ac_1h_rep1_S11_L004_R1_001.fastq.gz
H3K14ac_1h_Rep2.bam
H3K14ac_1h_rep2.bw
H3K14ac_1h_rep2_S36_L001_R1_001.fastq.gz
H3K14ac_1h_rep2_S36_L002_R1_001.fastq.gz
H3K14ac_1h_rep2_S36_L003_R1_001.fastq.gz
H3K14ac_1h_rep2_S36_L004_R1_001.fastq.gz

H3K14ac_3h.bam
H3K14ac_3h_peaks.bed
H3K14ac_3h_rep1.bw
H3K14ac_3h_rep1_S12_L001_R1_001.fastq.gz
H3K14ac_3h_rep1_S12_L002_R1_001.fastq.gz
H3K14ac_3h_rep1_S12_L003_R1_001.fastq.gz
H3K14ac_3h_rep1_S12_L004_R1_001.fastq.gz
H3K14ac_3h_Rep2.bam
H3K14ac_3h_rep2.bw
H3K14ac_3h_rep2_S34_L001_R1_001.fastq.gz
H3K14ac_3h_rep2_S34_L002_R1_001.fastq.gz
H3K14ac_3h_rep2_S34_L003_R1_001.fastq.gz
H3K14ac_3h_rep2_S34_L004_R1_001.fastq.gz
H3K14ac_6h.bam
H3K14ac_6h_peaks.bed
H3K14ac_6h_rep1.bw
H3K14ac_6h_rep1_S13_L001_R1_001.fastq.gz
H3K14ac_6h_rep1_S13_L002_R1_001.fastq.gz
H3K14ac_6h_rep1_S13_L003_R1_001.fastq.gz
H3K14ac_6h_rep1_S13_L004_R1_001.fastq.gz
H3K14ac_6h_Rep2.bam
H3K14ac_6h_rep2.bw
H3K14ac_6h_rep2_S33_L001_R1_001.fastq.gz
H3K14ac_6h_rep2_S33_L002_R1_001.fastq.gz
H3K14ac_6h_rep2_S33_L003_R1_001.fastq.gz
H3K14ac_6h_rep2_S33_L004_R1_001.fastq.gz
H3K27ac_0h.bam
H3K27ac_0h_Peaks.bed
H3K27ac_0h_rep1.bw
H3K27ac_0h_rep1_S2_L001_R1_001.fastq.gz
H3K27ac_0h_rep1_S2_L002_R1_001.fastq.gz
H3K27ac_0h_rep1_S2_L003_R1_001.fastq.gz
H3K27ac_0h_rep1_S2_L004_R1_001.fastq.gz
H3K27ac_0h_Rep2.bam
H3K27ac_0h_rep2.bw
H3K27ac_0h_rep2_S27_L001_R1_001.fastq.gz
H3K27ac_0h_rep2_S27_L002_R1_001.fastq.gz
H3K27ac_0h_rep2_S27_L003_R1_001.fastq.gz
H3K27ac_0h_rep2_S27_L004_R1_001.fastq.gz
H3K27ac_1h.bam
H3K27ac_1h_peaks.bed
H3K27ac_1h_rep1.bw
H3K27ac_1h_rep1_S3_L001_R1_001.fastq.gz
H3K27ac_1h_rep1_S3_L002_R1_001.fastq.gz
H3K27ac_1h_rep1_S3_L003_R1_001.fastq.gz
H3K27ac_1h_rep1_S3_L004_R1_001.fastq.gz
H3K27ac_1h_Rep2.bam
H3K27ac_1h_rep2.bw
H3K27ac_1h_rep2_S28_L001_R1_001.fastq.gz
H3K27ac_1h_rep2_S28_L002_R1_001.fastq.gz
H3K27ac_1h_rep2_S28_L003_R1_001.fastq.gz
H3K27ac_1h_rep2_S28_L004_R1_001.fastq.gz
H3K27ac_3h.bam
H3K27ac_3h_peaks.bed
H3K27ac_3h_rep1.bw
H3K27ac_3h_rep1_S4_L001_R1_001.fastq.gz
H3K27ac_3h_rep1_S4_L002_R1_001.fastq.gz
H3K27ac_3h_rep1_S4_L003_R1_001.fastq.gz
H3K27ac_3h_rep1_S4_L004_R1_001.fastq.gz
H3K27ac_3h_Rep2.bam
H3K27ac_3h_rep2.bw
H3K27ac_3h_rep2_S30_L001_R1_001.fastq.gz
H3K27ac_3h_rep2_S30_L002_R1_001.fastq.gz
H3K27ac_3h_rep2_S30_L003_R1_001.fastq.gz
H3K27ac_3h_rep2_S30_L004_R1_001.fastq.gz
H3K27ac_6h.bam
H3K27ac_6h_peaks.bed
H3K27ac_6h_rep1.bw
H3K27ac_6h_rep1_S5_L001_R1_001.fastq.gz
H3K27ac_6h_rep1_S5_L002_R1_001.fastq.gz
H3K27ac_6h_rep1_S5_L003_R1_001.fastq.gz
H3K27ac_6h_rep1_S5_L004_R1_001.fastq.gz
H3K27ac_6h_Rep2.bam
H3K27ac_6h_rep2.bw
H3K27ac_6h_rep2_S32_L001_R1_001.fastq.gz

H3K27ac_6h_rep2_S32_L002_R1_001.fastq.gz
H3K27ac_6h_rep2_S32_L003_R1_001.fastq.gz
H3K27ac_6h_rep2_S32_L004_R1_001.fastq.gz
H3K27me3_0h.bam
H3K27me3_0h_peaks.bed
H3K27me3_0h_rep1.bw
H3K27me3_0h_Rep2.bam
H3K27me3_0h_rep2.bw
H3K27me3_0h_rep2_S1_L001_R1_001.fastq.gz
H3K27me3_0h_rep2_S1_L002_R1_001.fastq.gz
H3K27me3_0h_rep2_S1_L003_R1_001.fastq.gz
H3K27me3_0h_rep2_S1_L004_R1_001.fastq.gz
H3K27me3_1h.bam
H3K27me3_1h_peaks.bed
H3K27me3_1h_rep1.bw
H3K27me3_1h_Rep2.bam
H3K27me3_1h_rep2.bw
H3K27me3_1h_rep2_S5_L001_R1_001.fastq.gz
H3K27me3_1h_rep2_S5_L002_R1_001.fastq.gz
H3K27me3_1h_rep2_S5_L003_R1_001.fastq.gz
H3K27me3_1h_rep2_S5_L004_R1_001.fastq.gz
H3K27me3_3h.bam
H3K27me3_3h_peaks.bed
H3K27me3_3h_rep1.bw
H3K27me3_3h_Rep2.bam
H3K27me3_3h_rep2.bw
H3K27me3_3h_rep2_S2_L001_R1_001.fastq.gz
H3K27me3_3h_rep2_S2_L002_R1_001.fastq.gz
H3K27me3_3h_rep2_S2_L003_R1_001.fastq.gz
H3K27me3_3h_rep2_S2_L004_R1_001.fastq.gz
H3K27me3_6h.bam
H3K27me3_6h_peaks.bed
H3K27me3_6h_rep1.bw
H3K27me3_6h_Rep2.bam
H3K27me3_6h_rep2.bw
H3K27me3_6h_rep2_S3_L001_R1_001.fastq.gz
H3K27me3_6h_rep2_S3_L002_R1_001.fastq.gz
H3K27me3_6h_rep2_S3_L003_R1_001.fastq.gz
H3K27me3_6h_rep2_S3_L004_R1_001.fastq.gz
H3K27me3_rep1_0h_S32_L001_R1_001.fastq.gz
H3K27me3_rep1_0h_S32_L002_R1_001.fastq.gz
H3K27me3_rep1_0h_S32_L003_R1_001.fastq.gz
H3K27me3_rep1_0h_S32_L004_R1_001.fastq.gz
H3K27me3_rep1_1h_S33_L001_R1_001.fastq.gz
H3K27me3_rep1_1h_S33_L002_R1_001.fastq.gz
H3K27me3_rep1_1h_S33_L003_R1_001.fastq.gz
H3K27me3_rep1_1h_S33_L004_R1_001.fastq.gz
H3K27me3_rep1_3h_S35_L001_R1_001.fastq.gz
H3K27me3_rep1_3h_S35_L002_R1_001.fastq.gz
H3K27me3_rep1_3h_S35_L003_R1_001.fastq.gz
H3K27me3_rep1_3h_S35_L004_R1_001.fastq.gz
H3K27me3_rep1_6h_S34_L001_R1_001.fastq.gz
H3K27me3_rep1_6h_S34_L002_R1_001.fastq.gz
H3K27me3_rep1_6h_S34_L003_R1_001.fastq.gz
H3K27me3_rep1_6h_S34_L004_R1_001.fastq.gz
H3K36me3_0h.bam
H3K36me3_0h_Peaks.bed
H3K36me3_0h_rep1.bw
H3K36me3_0h_rep1_S6_L001_R1_001.fastq.gz
H3K36me3_0h_rep1_S6_L002_R1_001.fastq.gz
H3K36me3_0h_rep1_S6_L003_R1_001.fastq.gz
H3K36me3_0h_rep1_S6_L004_R1_001.fastq.gz
H3K36me3_0h_Rep2.bam
H3K36me3_0h_rep2.bw
H3K36me3_0h_rep2_S1_L001_R1_001.fastq.gz
H3K36me3_0h_rep2_S1_L002_R1_001.fastq.gz
H3K36me3_0h_rep2_S1_L003_R1_001.fastq.gz
H3K36me3_0h_rep2_S1_L004_R1_001.fastq.gz
H3K36me3_1h.bam
H3K36me3_1h_peaks.bed
H3K36me3_1h_rep1.bw
H3K36me3_1h_rep1_S7_L001_R1_001.fastq.gz
H3K36me3_1h_rep1_S7_L002_R1_001.fastq.gz
H3K36me3_1h_rep1_S7_L003_R1_001.fastq.gz
H3K36me3_1h_rep1_S7_L004_R1_001.fastq.gz

H3K36me3_1h_Rep2.bam
H3K36me3_1h_rep2.bw
H3K36me3_1h_rep2_S7_L001_R1_001.fastq.gz
H3K36me3_1h_rep2_S7_L002_R1_001.fastq.gz
H3K36me3_1h_rep2_S7_L003_R1_001.fastq.gz
H3K36me3_1h_rep2_S7_L004_R1_001.fastq.gz
H3K36me3_3h.bam
H3K36me3_3h_peaks.bed
H3K36me3_3h_rep1.bw
H3K36me3_3h_rep1_S8_L001_R1_001.fastq.gz
H3K36me3_3h_rep1_S8_L002_R1_001.fastq.gz
H3K36me3_3h_rep1_S8_L003_R1_001.fastq.gz
H3K36me3_3h_rep1_S8_L004_R1_001.fastq.gz
H3K36me3_3h_Rep2.bam
H3K36me3_3h_rep2.bw
H3K36me3_3h_rep2_S8_L001_R1_001.fastq.gz
H3K36me3_3h_rep2_S8_L002_R1_001.fastq.gz
H3K36me3_3h_rep2_S8_L003_R1_001.fastq.gz
H3K36me3_3h_rep2_S8_L004_R1_001.fastq.gz
H3K36me3_6h.bam
H3K36me3_6h_peaks.bed
H3K36me3_6h_rep1.bw
H3K36me3_6h_rep1_S9_L001_R1_001.fastq.gz
H3K36me3_6h_rep1_S9_L002_R1_001.fastq.gz
H3K36me3_6h_rep1_S9_L003_R1_001.fastq.gz
H3K36me3_6h_rep1_S9_L004_R1_001.fastq.gz
H3K36me3_6h_Rep2.bam
H3K36me3_6h_rep2.bw
H3K36me3_6h_rep2_S4_L001_R1_001.fastq.gz
H3K36me3_6h_rep2_S4_L002_R1_001.fastq.gz
H3K36me3_6h_rep2_S4_L003_R1_001.fastq.gz
H3K36me3_6h_rep2_S4_L004_R1_001.fastq.gz
H3K4me3_0h.bam
H3K4me3_0h_Peaks.bed
H3K4me3_0h_rep1.bw
H3K4me3_0h_rep1_S14_L001_R1_001.fastq.gz
H3K4me3_0h_rep1_S14_L002_R1_001.fastq.gz
H3K4me3_0h_rep1_S14_L003_R1_001.fastq.gz
H3K4me3_0h_rep1_S14_L004_R1_001.fastq.gz
H3K4me3_0h_Rep2.bam
H3K4me3_0h_rep2.bw
H3K4me3_0h_rep2_S5_L001_R1_001.fastq.gz
H3K4me3_0h_rep2_S5_L002_R1_001.fastq.gz
H3K4me3_0h_rep2_S5_L003_R1_001.fastq.gz
H3K4me3_0h_rep2_S5_L004_R1_001.fastq.gz
H3K4me3_1h.bam
H3K4me3_1h_peaks.bed
H3K4me3_1h_rep1.bw
H3K4me3_1h_rep1_S17_L001_R1_001.fastq.gz
H3K4me3_1h_rep1_S17_L002_R1_001.fastq.gz
H3K4me3_1h_rep1_S17_L003_R1_001.fastq.gz
H3K4me3_1h_rep1_S17_L004_R1_001.fastq.gz
H3K4me3_1h_Rep2.bam
H3K4me3_1h_rep2.bw
H3K4me3_1h_rep2_S6_L001_R1_001.fastq.gz
H3K4me3_1h_rep2_S6_L002_R1_001.fastq.gz
H3K4me3_1h_rep2_S6_L003_R1_001.fastq.gz
H3K4me3_1h_rep2_S6_L004_R1_001.fastq.gz
H3K4me3_3h.bam
H3K4me3_3h_peaks.bed
H3K4me3_3h_rep1.bw
H3K4me3_3h_rep1_S20_L001_R1_001.fastq.gz
H3K4me3_3h_rep1_S20_L002_R1_001.fastq.gz
H3K4me3_3h_rep1_S20_L003_R1_001.fastq.gz
H3K4me3_3h_rep1_S20_L004_R1_001.fastq.gz
H3K4me3_3h_Rep2.bam
H3K4me3_3h_rep2.bw
H3K4me3_3h_rep2_S31_L001_R1_001.fastq.gz
H3K4me3_3h_rep2_S31_L002_R1_001.fastq.gz
H3K4me3_3h_rep2_S31_L003_R1_001.fastq.gz
H3K4me3_3h_rep2_S31_L004_R1_001.fastq.gz
H3K4me3_6h.bam
H3K4me3_6h_peaks.bed
H3K4me3_6h_rep1.bw
H3K4me3_6h_rep1_S23_L001_R1_001.fastq.gz

H3K4me3_6h_rep1_S23_L002_R1_001.fastq.gz
H3K4me3_6h_rep1_S23_L003_R1_001.fastq.gz
H3K4me3_6h_rep1_S23_L004_R1_001.fastq.gz
H3K4me3_6h_Rep2.bam
H3K4me3_6h_rep2.bw
H3K4me3_6h_rep2_S29_L001_R1_001.fastq.gz
H3K4me3_6h_rep2_S29_L002_R1_001.fastq.gz
H3K4me3_6h_rep2_S29_L003_R1_001.fastq.gz
H3K4me3_6h_rep2_S29_L004_R1_001.fastq.gz

For the MB3 data set (E-MTAB-8265) the following files were uploaded:
H3_0h.bam
H3_0h_rep1_S1_L002_R1_001.fastq.gz
H3_0h_rep1_S1_L003_R1_001.fastq.gz
H3_0h_rep1_S1_L004_R1_001.fastq.gz
H3_0h_Rep2.bam
H3_0h_rep2_S1_L001_R1_001.fastq.gz
H3_0h_rep2_S1_L002_R1_001.fastq.gz
H3_0h_rep2_S1_L003_R1_001.fastq.gz
H3_0h_rep2_S1_L004_R1_001.fastq.gz
H3_1h.bam
H3_1h_rep1_S16_L001_R1_001.fastq.gz
H3_1h_rep1_S16_L002_R1_001.fastq.gz
H3_1h_rep1_S16_L003_R1_001.fastq.gz
H3_1h_rep1_S16_L004_R1_001.fastq.gz
H3_1h_Rep2.bam
H3_1h_rep2_S3_L001_R1_001.fastq.gz
H3_1h_rep2_S3_L002_R1_001.fastq.gz
H3_1h_rep2_S3_L003_R1_001.fastq.gz
H3_1h_rep2_S3_L004_R1_001.fastq.gz
H3_3h.bam
H3_3h_rep1_S19_L001_R1_001.fastq.gz
H3_3h_rep1_S19_L002_R1_001.fastq.gz
H3_3h_rep1_S19_L003_R1_001.fastq.gz
H3_3h_rep1_S19_L004_R1_001.fastq.gz
H3_3h_Rep2.bam
H3_3h_rep2_S2_L001_R1_001.fastq.gz
H3_3h_rep2_S2_L002_R1_001.fastq.gz
H3_3h_rep2_S2_L003_R1_001.fastq.gz
H3_3h_rep2_S2_L004_R1_001.fastq.gz
H3_6h.bam
H3_6h_rep1_S22_L001_R1_001.fastq.gz
H3_6h_rep1_S22_L002_R1_001.fastq.gz
H3_6h_rep1_S22_L003_R1_001.fastq.gz
H3_6h_rep1_S22_L004_R1_001.fastq.gz
H3_6h_Rep2.bam
H3_6h_rep2_S4_L001_R1_001.fastq.gz
H3_6h_rep2_S4_L002_R1_001.fastq.gz
H3_6h_rep2_S4_L003_R1_001.fastq.gz
H3_6h_rep2_S4_L004_R1_001.fastq.gz
H3K14ac_0h.bam
H3K14ac_0h_Peaks.bed
H3K14ac_0h_rep1.bw
H3K14ac_0h_rep1_S10_L001_R1_001.fastq.gz
H3K14ac_0h_rep1_S10_L002_R1_001.fastq.gz
H3K14ac_0h_rep1_S10_L003_R1_001.fastq.gz
H3K14ac_0h_rep1_S10_L004_R1_001.fastq.gz
H3K14ac_0h_Rep2.bam
H3K14ac_0h_rep2.bw
H3K14ac_0h_rep2_S35_L001_R1_001.fastq.gz
H3K14ac_0h_rep2_S35_L002_R1_001.fastq.gz
H3K14ac_0h_rep2_S35_L003_R1_001.fastq.gz
H3K14ac_0h_rep2_S35_L004_R1_001.fastq.gz
H3K14ac_1h.bam
H3K14ac_1h_peaks.bed
H3K14ac_1h_rep1.bw
H3K14ac_1h_rep1_S11_L001_R1_001.fastq.gz
H3K14ac_1h_rep1_S11_L002_R1_001.fastq.gz
H3K14ac_1h_rep1_S11_L003_R1_001.fastq.gz
H3K14ac_1h_rep1_S11_L004_R1_001.fastq.gz
H3K14ac_1h_Rep2.bam
H3K14ac_1h_rep2.bw
H3K14ac_1h_rep2_S36_L001_R1_001.fastq.gz
H3K14ac_1h_rep2_S36_L002_R1_001.fastq.gz
H3K14ac_1h_rep2_S36_L003_R1_001.fastq.gz

H3K14ac_1h_rep2_S36_L004_R1_001.fastq.gz
H3K14ac_3h.bam
H3K14ac_3h_peaks.bed
H3K14ac_3h_rep1.bw
H3K14ac_3h_rep1_S12_L001_R1_001.fastq.gz
H3K14ac_3h_rep1_S12_L002_R1_001.fastq.gz
H3K14ac_3h_rep1_S12_L003_R1_001.fastq.gz
H3K14ac_3h_rep1_S12_L004_R1_001.fastq.gz
H3K14ac_3h_Rep2.bam
H3K14ac_3h_rep2.bw
H3K14ac_3h_rep2_S34_L001_R1_001.fastq.gz
H3K14ac_3h_rep2_S34_L002_R1_001.fastq.gz
H3K14ac_3h_rep2_S34_L003_R1_001.fastq.gz
H3K14ac_3h_rep2_S34_L004_R1_001.fastq.gz
H3K14ac_6h.bam
H3K14ac_6h_peaks.bed
H3K14ac_6h_rep1.bw
H3K14ac_6h_rep1_S13_L001_R1_001.fastq.gz
H3K14ac_6h_rep1_S13_L002_R1_001.fastq.gz
H3K14ac_6h_rep1_S13_L003_R1_001.fastq.gz
H3K14ac_6h_rep1_S13_L004_R1_001.fastq.gz
H3K14ac_6h_Rep2.bam
H3K14ac_6h_rep2.bw
H3K14ac_6h_rep2_S33_L001_R1_001.fastq.gz
H3K14ac_6h_rep2_S33_L002_R1_001.fastq.gz
H3K14ac_6h_rep2_S33_L003_R1_001.fastq.gz
H3K14ac_6h_rep2_S33_L004_R1_001.fastq.gz
H3K4me3_0h.bam
H3K4me3_0h_Peaks.bed
H3K4me3_0h_rep1.bw
H3K4me3_0h_rep1_S14_L001_R1_001.fastq.gz
H3K4me3_0h_rep1_S14_L002_R1_001.fastq.gz
H3K4me3_0h_rep1_S14_L003_R1_001.fastq.gz
H3K4me3_0h_rep1_S14_L004_R1_001.fastq.gz
H3K4me3_0h_Rep2.bam
H3K4me3_0h_rep2.bw
H3K4me3_0h_rep2_S5_L001_R1_001.fastq.gz
H3K4me3_0h_rep2_S5_L002_R1_001.fastq.gz
H3K4me3_0h_rep2_S5_L003_R1_001.fastq.gz
H3K4me3_0h_rep2_S5_L004_R1_001.fastq.gz
H3K4me3_1h.bam
H3K4me3_1h_peaks.bed
H3K4me3_1h_rep1.bw
H3K4me3_1h_rep1_S17_L001_R1_001.fastq.gz
H3K4me3_1h_rep1_S17_L002_R1_001.fastq.gz
H3K4me3_1h_rep1_S17_L003_R1_001.fastq.gz
H3K4me3_1h_rep1_S17_L004_R1_001.fastq.gz
H3K4me3_1h_Rep2.bam
H3K4me3_1h_rep2.bw
H3K4me3_1h_rep2_S6_L001_R1_001.fastq.gz
H3K4me3_1h_rep2_S6_L002_R1_001.fastq.gz
H3K4me3_1h_rep2_S6_L003_R1_001.fastq.gz
H3K4me3_1h_rep2_S6_L004_R1_001.fastq.gz
H3K4me3_3h.bam
H3K4me3_3h_peaks.bed
H3K4me3_3h_rep1.bw
H3K4me3_3h_rep1_S20_L001_R1_001.fastq.gz
H3K4me3_3h_rep1_S20_L002_R1_001.fastq.gz
H3K4me3_3h_rep1_S20_L003_R1_001.fastq.gz
H3K4me3_3h_rep1_S20_L004_R1_001.fastq.gz
H3K4me3_3h_Rep2.bam
H3K4me3_3h_rep2.bw
H3K4me3_3h_rep2_S31_L001_R1_001.fastq.gz
H3K4me3_3h_rep2_S31_L002_R1_001.fastq.gz
H3K4me3_3h_rep2_S31_L003_R1_001.fastq.gz
H3K4me3_3h_rep2_S31_L004_R1_001.fastq.gz
H3K4me3_6h.bam
H3K4me3_6h_peaks.bed
H3K4me3_6h_rep1.bw
H3K4me3_6h_rep1_S23_L001_R1_001.fastq.gz
H3K4me3_6h_rep1_S23_L002_R1_001.fastq.gz
H3K4me3_6h_rep1_S23_L003_R1_001.fastq.gz
H3K4me3_6h_rep1_S23_L004_R1_001.fastq.gz
H3K4me3_6h_Rep2.bam
H3K4me3_6h_rep2.bw

H3K4me3_6h_rep2_S29_L001_R1_001.fastq.gz
H3K4me3_6h_rep2_S29_L002_R1_001.fastq.gz
H3K4me3_6h_rep2_S29_L003_R1_001.fastq.gz
H3K4me3_6h_rep2_S29_L004_R1_001.fastq.gz

Genome browser session (e.g. UCSC)

At the moment, it is not provided, since for Arabidopsis, we are not aware of a platform to anonymously and privately share our viewer data. We therefore added BigWig files in the data deposition that allows viewing the peaks in genome viewer (IGV; JBrowse etc.) by simply uploading the files.

## Methodology

Replicates

ChIP-SEQ was perfomed in duplicate and only peaks in both replicates were taken into account for further analysis.

Sequencing depth

All reads were single end and 50 bp in length

For the wounding data set (E-MTAB-7611) read depth:

H3_0h.bam # reads processed: 21968291 # reads with at least one reported alignment: 16502277 (75.12%)
H3_0h_Rep2.bam # reads processed: 22274641 # reads with at least one reported alignment: 16163234 (72.56%)
H3_1h.bam # reads processed: 35618989 # reads with at least one reported alignment: 27112675 (76.12%)
H3_1h_Rep2.bam # reads processed: 26717609 # reads with at least one reported alignment: 19118347 (71.56%)
H3_3h.bam # reads processed: 33281598 # reads with at least one reported alignment: 24905204 (74.83%)
H3_3h_Rep2.bam # reads processed: 23583858 # reads with at least one reported alignment: 16832047 (71.37%)
H3_6h.bam # reads processed: 38031357 # reads with at least one reported alignment: 28133076 (73.97%)
H3_6h_Rep2.bam # reads processed: 20849216 # reads with at least one reported alignment: 15115526 (72.50%)
H3K14ac_0h.bam # reads processed: 24810582 # reads with at least one reported alignment: 19001252 (76.59%)
H3K14ac_0h_Rep2.bam # reads processed: 23010624 # reads with at least one reported alignment: 19155415 (83.25%)
H3K14ac_1h.bam # reads processed: 22185301 # reads with at least one reported alignment: 15741808 (70.96%)
H3K14ac_1h_Rep2.bam # reads processed: 17746225 # reads with at least one reported alignment: 14340130 (80.81%)
H3K14ac_3h.bam # reads processed: 25025441 # reads with at least one reported alignment: 18874834 (75.42%)
H3K14ac_3h_Rep2.bam # reads processed: 22609345 # reads with at least one reported alignment: 18497675 (81.81%)
H3K14ac_6h.bam # reads processed: 23058474 # reads with at least one reported alignment: 15878034 (68.86%)
H3K14ac_6h_Rep2.bam # reads processed: 19142340 # reads with at least one reported alignment: 15939489 (83.27%)
H3K27ac_0h.bam # reads processed: 22200449 # reads with at least one reported alignment: 16990801 (76.53%)
H3K27ac_0h_Rep2.bam # reads processed: 25186035 # reads with at least one reported alignment: 20778478 (82.50%)
H3K27ac_1h.bam # reads processed: 23508642 # reads with at least one reported alignment: 19245562 (81.87%)
H3K27ac_1h_Rep2.bam # reads processed: 27681352 # reads with at least one reported alignment: 22264225 (80.43%)
H3K27ac_3h.bam # reads processed: 26215292 # reads with at least one reported alignment: 19410069 (74.04%)
H3K27ac_3h_Rep2.bam # reads processed: 25622425 # reads with at least one reported alignment: 20285021 (79.17%)
H3K27ac_6h.bam # reads processed: 24243423 # reads with at least one reported alignment: 19443968 (80.20%)
H3K27ac_6h_Rep2.bam # reads processed: 24150983 # reads with at least one reported alignment: 19877573 (82.31%)
H3K27me3_0h.bam # reads processed: 20378560 # reads with at least one reported alignment: 14571788 (71.51%)
H3K27me3_0h_Rep2.bam # reads processed: 12004967 # reads with at least one reported alignment: 9322857 (77.66%)
H3K27me3_1h.bam # reads processed: 25441622 # reads with at least one reported alignment: 18414306 (72.38%)
H3K27me3_1h_Rep2.bam # reads processed: 19156359 # reads with at least one reported alignment: 13916508 (72.65%)
H3K27me3_3h.bam # reads processed: 17577702 # reads with at least one reported alignment: 13194679 (75.06%)
H3K27me3_3h_Rep2.bam # reads processed: 12411374 # reads with at least one reported alignment: 9382439 (75.60%)
H3K27me3_6h.bam # reads processed: 20467401 # reads with at least one reported alignment: 15035251 (73.46%)
H3K27me3_6h_Rep2.bam # reads processed: 14619381 # reads with at least one reported alignment: 11022996 (75.40%)
H3K36me3_0h.bam # reads processed: 29456271 # reads with at least one reported alignment: 24578995 (83.44%)
H3K36me3_0h_Rep2.bam # reads processed: 8852031 # reads with at least one reported alignment: 7817962 (88.32%)
H3K36me3_1h.bam # reads processed: 22563987 # reads with at least one reported alignment: 17858432 (79.15%)
H3K36me3_1h_Rep2.bam # reads processed: 12174701 # reads with at least one reported alignment: 10737212 (88.19%)
H3K36me3_3h.bam # reads processed: 36316368 # reads with at least one reported alignment: 29013106 (79.89%)
H3K36me3_3h_Rep2.bam # reads processed: 34460979 # reads with at least one reported alignment: 30630948 (88.89%)
H3K36me3_6h.bam # reads processed: 27614030 # reads with at least one reported alignment: 23797379 (86.18%)
H3K36me3_6h_Rep2.bam # reads processed: 19129534 # reads with at least one reported alignment: 16960599 (88.66%)
H3K4me3_0h.bam # reads processed: 27664295 # reads with at least one reported alignment: 24018497 (86.82%)
H3K4me3_0h_Rep2.bam # reads processed: 23423360 # reads with at least one reported alignment: 21547710 (91.99%)
H3K4me3_1h.bam # reads processed: 22867732 # reads with at least one reported alignment: 19875197 (86.91%)
H3K4me3_1h_Rep2.bam # reads processed: 21224092 # reads with at least one reported alignment: 19447993 (91.63%)
H3K4me3_3h.bam # reads processed: 37469915 # reads with at least one reported alignment: 31850836 (85.00%)
H3K4me3_3h_Rep2.bam # reads processed: 26987832 # reads with at least one reported alignment: 24746500 (91.70%)
H3K4me3_6h.bam # reads processed: 37277914 # reads with at least one reported alignment: 33252948 (89.20%)
H3K4me3_6h_Rep2.bam # reads processed: 18946075 # reads with at least one reported alignment: 17310914 (91.37%)

For the MB3g data set (E-MTAB-8265) read depth:

MB3_H3_0h_post-wounding.bam # reads processed: 10791851 # reads with at least one reported alignment: 9619466 (89.14%)
MB3_H3_3h_post-wounding.bam # reads processed: 15548359 # reads with at least one reported alignment: 13976604 (89.89%)
MB3_H3_6h_post-wounding.bam # reads processed: 12537400 # reads with at least one reported alignment: 10754710 (85.78%)

MB3_H3K14ac_0h_post-wounding.bam # reads processed: 12830112 # reads with at least one reported alignment: 9276167 (72.30%)
MB3_H3K14ac_3h_post-wounding.bam # reads processed: 15063386 # reads with at least one reported alignment: 9544528 (63.36%)
MB3_H3K14ac_6h_post-wounding.bam # reads processed: 14183338 # reads with at least one reported alignment: 9629079 (67.89%)
MB3_H3K4me3_0h_post-wounding.bam bam # reads processed: 16052181 # reads with at least one reported alignment: 13270673 (82.67%)
MB3_H3K4me3_3h_post-wounding.bam bam # reads processed: 18345209 # reads with at least one reported alignment: 14595608 (79.56%)
MB3_H3K4me3_6h_post-wounding.bam bam # reads processed: 15155293 # reads with at least one reported alignment: 12188521 (80.43%)

Antibodies

H3 (ab1791; Abcam), H3K27me3 (07-449; Millipore), H3K4me3 (ab8580; Abcam), H3K36me3 (ab9050; Abcam), H3K9/14ac (06-599, Millipore) and H3K27ac (ab4729, Abcam).

Peak calling parameters

MACS2 settings were used for H3K4me3, H3K9/14ac and H3K27ac, and the "–broad" option was used for H3K36me3 and H3K27me3. H3 peaks were used as control for the baseline, and only peaks with Q < 0.001 were considered. finally only peaks in both duplicates were kept for further analysis.

Data quality

Quality of data was checked using standard FASTQC Quality Control. To ensure processing of reliable data only peaks found in both replicates were considered with a Q value cut off of 0.001. Considered peaks: H3K27me3: 6085 peaks, H3K4me3: 14295 peaks, H3K36me3: 12617 peaks, H3K27ac: 14312 peaks, H3K9/14ac: 12996 peaks.

Software

Mapping using Bowtie, keeping only the reads mapped to a single unique position, MACS2 for peak calling, BEDTools for file conversions and peak annotation, R-Studio for data analysis using the MAnorm normalization for identification of peaks with altered profiles. Deeptools and IGV viewer for data vizualization.
